# Correlation Between Amygdala Nuclei Volumes and Memory in Cognitively Normal Adults Carrying the ApoE ε3/ε3 Allele

**DOI:** 10.3389/fnagi.2021.747288

**Published:** 2021-12-13

**Authors:** Wenqing Liao, Dong Cui, Jingna Jin, Wenbo Liu, Xin Wang, He Wang, Ying Li, Zhipeng Liu, Tao Yin

**Affiliations:** ^1^Institute of Biomedical Engineering, Chinese Academy of Medical Sciences and Peking Union Medical College, Tianjin, China; ^2^Sinovation (Beijing) Medical Technology Co., Ltd., Beijing, China; ^3^Neuroscience Center, Chinese Academy of Medical Sciences, Beijing, China

**Keywords:** amygdala nuclei, aging, ApoE, immediate recall, delayed recall, delayed recognition

## Abstract

The amygdala is known to be related to cognitive function. In this study, we used an automated approach to segment the amygdala into nine nuclei and evaluated amygdala and nuclei volumetric changes across the adult lifespan in subjects carrying the apolipoprotein E (ApoE) ε3/ε3 allele, and we related those changes to memory function alteration. We found that except the left medial nucleus (Me), whose volume decreased in the old group compared with the middle-early group, all other nuclei volumes presented a significant decline in the old group compared with the young group. Left accessory basal nucleus (AB) and left cortico-amygdaloid transition area (CAT) volumes were also diminished in the middle-late group. In addition, immediate memory recall is impaired by the process of aging, whereas delayed recall and delayed recognition memory functions were not significantly changed. We found significant positive correlations between immediate recall scores and volumes of the bilateral basal nucleus (Ba), AB, anterior amygdaloid area (AAA), CAT, whole amygdala, left lateral nucleus (La), left paralaminar nucleus (PL), and right cortical nucleus (Co). The results suggest that immediate recall memory decline might be associated with volumetric reduction of the amygdala and its nuclei, and the left AB and left CAT might be considered as potential imaging biomarkers of memory decline in aging.

## Introduction

The amygdala is a prominent limbic formation and plays a key role in emotional and cognitive processes (AbuHasan et al., 2020). Dysfunction of the amygdala has been implicated in a number of different neurodevelopmental disorders and psychopathologies (Davis and Whalen, 2001; Belkhiria et al., 2020; Cui et al., 2020), such as depression (Abercrombie et al., 1998), social anxiety disorder (Klumpp and Fitzgerald, 2018), post-traumatic stress disorder (Rauch et al., 2000), dementia (Cavedo et al., 2011), and schizophrenia (Prestia et al., 2011). Previous studies have shown that the amygdala is also involved in advanced cognitive abilities (Belkhiria et al., 2020; Li et al., 2021), such as memory (Adolphs et al., 1997; McIntyre et al., 2003; Schaefer et al., 2006; Taujanskaitė et al., 2020), learning (Fried et al., 2001; Aquino et al., 2020), decision-making (Bechara et al., 2003), reward behavior (Sharp, 2019), and intelligence (Gray et al., 2003; Li et al., 2021). In recent years, more and more studies have reported that the amygdala is associated with memory function, such as emotional memory (Dolcos et al., 2017), memory consolidation (Huff et al., 2013; Lalumiere, 2014), working memory (Fried et al., 2001; McIntyre et al., 2003; Schaefer et al., 2006), state-dependent memory (Baidoo et al., 2020), autobiographical memory (Young et al., 2017), and episodic memory (Kensinger et al., 2011). Most studies have treated the amygdala as a whole structure. However, the amygdala is composed of multiple nuclei with unique functions and connections in the limbic system and to the rest of the brain. Hence, it is possible that amygdala nuclei may differ from each other in age-related volumetric changes and their relation to memory.

Age and apolipoprotein E (ApoE) are the mightiest risk factors for Alzheimer’s disease (AD), but the underlying mechanisms remain unclear. In human, ApoE is expressed by the polymorphic alleles: ε2, ε3, and ε4. The ε4 allele is the most risky gene for AD, ε2 allele may provide a protective effect, and ε3 is the most common allele in all human populations, at frequencies ranging from 69 to 85% (Belloy et al., 2019). Evidence shows that amygdala nuclei volumes are affected by ApoE genotype. For example, a study (Aghamohammadi-Sereshki et al., 2019) segmented the amygdala manually and compared amygdala nuclei volumes between healthy younger (18–54 years) and older (≥55 years) carriers of the same ApoE allele. They found smaller lateral, basal, and accessory basal nuclei and total amygdala volume, among older ApoE ε3 and ApoE ε4 allele carriers compared to their younger counterparts, while older ApoE ε2 allele carriers did not differ in any amygdala nuclei volumes from younger counterparts. Furthermore, they found that the effect size of age-related volumetric differences was the largest among the ApoE ε4 carriers. To date, few studies have studied the association between amygdala nuclei and memory function, especially with the ApoE genotype taken into consideration.

The objective of this study is to describe age-related volumetric growth and/or decline of the amygdala and its nuclei structures across the human adult lifespan. Furthermore, we examined the relationship between the amygdala and its nuclei volume and memory function. We hypothesized that specific nuclei of the amygdala would be associated with memory recall scores. To this end, we used a cross-sectional sample of 315 individuals, aged from 20 to 89 years, to investigate different stages of the adult lifespan with respect to alterations in amygdala and its nuclei volume and memory function. Simultaneously, in order to eliminate the potential impact of ApoE, only ApoE ε3/ε3 allele carriers were analyzed.

## Materials and Methods

### Participants

A total of 315 healthy adults, aged 20-89 years, were selected from the Dallas Lifespan Brain Study (DLBS).^1^ The inclusion and exclusion criteria were as follows:

Inclusion criteria: (1) right-handed and native English speakers, (2) without a history of neurological disease, (3) well-educated and cognitively normal as measured with the Mini-Mental State Examination (MMSE > 26), and (4) with high-quality sMRI data.

Exclusion criteria: (1) No genotype information, (2) ApoE ε2 or ε4 carriers, and (3) incomplete cognitive tests. Therefore, 70 subjects with no genotype information, 56 ApoE ε2 or ε4 carriers (ε2/ε2 = 2, ε2/ε3 = 12, ε2/ε4 = 5, ε4/ε3 = 32, ε4/ε4 = 5), 40 subjects with incomplete cognitive tests were excluded.

Finally, a total of 149 subjects (58.49 ± 19.81 years; 90 females, 59 males) were included in the present study. The subjects were classified into four groups: Young group (20–35 years, F/M = 17/12), Middle-early group (36–50 years, F/M = 11/12), Middle-late group (51–65 years, F/M = 23/11), and Old group (66–89 years, F/M = 39/24).

### Neuropsychological Assessment for Memory Function

In the present study, participants went through the Hopkins verbal learning test and the Cambridge Neuropsychological Test Automated Battery verbal recognition memory (CANTAB_VRM) test for memory assessment. The Hopkins verbal learning test is composed of three consecutive tasks for immediate recall, delayed recall, and delayed recognition. First, participants listened to a list of 12 words, and then they were asked to recall as many words from the list as they could. Twenty minutes later, participants were asked again to recall as many words as they could remember. After that, participants listened to a new list of 24 words and had to determine if the words were part of the initial list. The three scores were as follows: HOP immediate recall, number of words correctly recalled; HOP delayed recall, number of words correctly recalled after a 20-min delay; HOP recognition, number of items correctly identified as “old” or “new” in delayed recognition.

The CANTAB_VRM task also assesses immediate recall. In this task, the participants were shown a list of 12 words and asked to read each word aloud one at a time. Immediately after presentation, participants were asked to recall as many words as they could remember. The task was scored on the total number of words remembered.

### Structural MRI Data Acquisition

All participants underwent T1-weighted imaging in a Philips Achieva 3T scanner (Amsterdam, Netherlands). The MRI data acquired were 160 sagittal slice high-resolution T1-weighted images using magnetization-prepared rapid gradient-echo (MP-RAGE) sequences with a voxel size of 1 mm^3^. The parameters were as follows: slice thickness = 1 mm, repetition time (TR) = 8.135 ms, echo time (TE) = 3.7 ms, flip angle = 12°, matrix = 256 × 256, field of view (FOV) = 204 × 256.

### Imaging Processing

**FIGURE 1:**
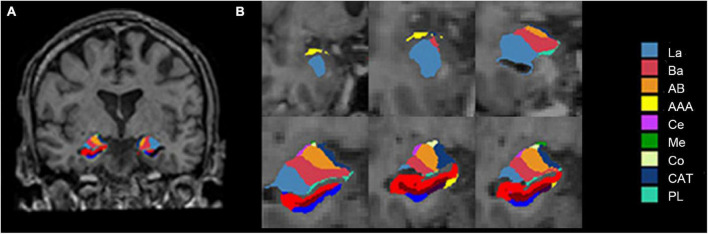
Amygdala nuclei segmentation: **(A)** Amygdala nuclei segmentation in coronal view; **(B)** enlarged view of the right amygdala segmentation. La, lateral nucleus; Ba, basal nucleus; AB, accessory basal nucleus; Ce, central nucleus; Me, medial nucleus; Co, cortical nucleus; AAA, anterior amygdaloid area; CAT, cortico-amygdaloid transition area; PL, paralaminar nucleus; Whole, whole amygdala.

In this study, volumetric segmentation was performed with the FreeSurfer image analysis software (version 7.1.1).^2^ The “recon-all” processing stream with default parameters was used for subcortical volume analysis. Details of the segmentation methods and procedures are described in prior publications (Fischl et al., 2002; Jayakar et al., 2020). Briefly, the T1-weighted image was segmented into gray matter, white matter, and cerebrospinal fluid. Subsequently, the segmentation of subcortical structures was examined by a non-linear warping atlas, yielding volumetric measures of deep gray matter, including the thalamus, caudate, putamen, amygdala, hippocampus, pallidum, and accumbens. Furthermore, the amygdala subnuclei segmentation module was used to parcellate the hippocampus, amygdala, and thalamus subnuclei further. A probabilistic atlas and a modified version of Van Leemput’s algorithm (Iglesias et al., 2015) was applied on the segmentation of amygdala (Saygin et al., 2017). Ultimately, the amygdala was divided into nine nuclei—the lateral nucleus (La), basal nucleus (Ba), accessory basal nucleus (AB), central nucleus (Ce), medial nucleus (Me), cortical nucleus (Co), anterior amygdaloid area (AAA), cortico-amygdaloid transition area (CAT), and paralaminar nucleus (PL), as shown in Figure 1. Volumes were visually inspected for misclassifications during the reconstruction process.

### Statistical Analysis

Statistical analysis was performed using SPSS software (version 21.0, IBM, Armonk, NY, United States). The chi-square test was used to evaluate the differences in gender among four groups. The Kruskal-Wallis test was used to analyze group differences in education years. Amygdala nuclei volumes and cognitive tests scores were standardized using Z-score method. Analysis of covariance (ANCOVA) was performed for group differences in MMSE and memory tests scores, with gender and education years as covariates. To examine amygdala nuclei volume group differences, we performed ANCOVA with gender, education years, and estimated total intracranial volume (eTIV) as covariates. Pairwise comparisons using the Bonferroni method were performed for indexes with significant group differences. Spearman correlation analysis was applied to examine the correlations between memory scores and amygdala nuclei volumes across the life span, *P*-values and correlation coefficients (r) were calculated, with gender, education years, and eTIV regarded as covariates. Correlation results were corrected by the false discovery rate (FDR). Meanwhile, we tested the mediating effect of the hippocampus in the relationship between amygdala nuclei volumes and memory function, using single-mediator model with gender, education years and eTIV as covariates. The significance level for all results was set at *P* < 0.05.

## Results

### Differences in Demographics and Cognitive Test Scores

Description and analysis of demographic characteristics for the 149 subjects are shown in Table 1. There was no significant difference among groups in gender or education years. MMSE scores, although still within the normal range, significantly declined with age (*P* < 0.001). Pairwise comparison results are detailed in Supplementary Table 1. An apparent decline of MMSE scores was observed in the Old group compared with the other three groups.

**TABLE 1:** Demographic information.

Characteristics	Young	Middle-early	Middle-late	Old	χ^2*a*^/F*^b^*	*P* *^b^*	*F* *^c^*	*P* *^c^*
	(*n* = 29)	(*n* = 23)	(*n* = 34)	(*n* = 63)				
Age (years)	28.4 ± 4.24	43.93 ± 4.77	59.22 ± 4.51	77.27 ± 6.91	n.d.	n.d.	n.d.	n.d.
Gender (F/M)	17/12	11/12	23/11	39/24	2.365^a^	0.500	n.d.	n.d.
Education (years)	16.64 ± 1.97	16.46 ± 2.31	16.24 ± 2.20	16.01 ± 2.36	1.328^a^	0.722	n.d.	n.d.
MMSE	28.76 ± 1.15	28.57 ± 1.16	28.65 ± 1.07	27.63 ± 1.29	9.071	<0.001***	8.823	<0.001***

Data expressed as mean ± SD; MMSE, Mini-Mental State Exam; n.d., not done; ***P < 0.001.

*^a^Chi-square test.*

*^b^ANOVA, no covariates.*

*^c^ANCOVA, controlling for gender and education years.*

### Differences in Cerebral Compartment Volumes

Description and analysis of the cerebral compartment volumes for the 149 subjects are shown in Table 2. No significant group difference was observed for eTIV. Apparent volumetric decline was observed for gray matter, white matter and cerebrospinal fluid volumes. The gray matter volume declined along aging, cerebrospinal fluid volumes declined in the Middle-late and Old groups, and white matter volumes declined only in the Old group. Detailed pairwise comparison and scatter plots are presented in Supplementary Table 2 and Supplementary Figure 1, respectively.

**TABLE 2:** Cerebral compartment volumes.

Compartment (cm^3^)	Young	Middle-early	Middle-late	Old	*F* *^a^*	*P* *^a^*	*F^b^*,*^c^*	*P^b^*,*^c^*
	(*n* = 29)	(*n* = 23)	(*n* = 34)	(*n* = 63)				
eTIV	1408.21 ± 17.83	1394.82 ± 20.07	1381.02 ± 16.46	1429.89 ± 12.09	1.608	0.190	2.129^b^	0.099^b^
Gray matter	673.59 ± 9.23	633.32 ± 5.81	607.31 ± 10.36	593.79 ± 8.52	40.88	< 0.001***	143.00	< 0.001***
White matter	533.74 ± 10.92	539.94 ± 12.26	508.63 ± 10.08	488.61 ± 7.41	6.35	< 0.001***	24.49	< 0.001***
Cerebro-spinal fluid	224.22 ± 13.58	269.67 ± 15.25	308.46 ± 12.54	429.08 ± 9.21	64.49	< 0.001***	103.65	< 0.001***

Data expressed as mean ± SD; eTIV, estimated total intracranial volume; n.d., not done; ***P < 0.001.

*^a^ANOVA, no covariates.*

*^b^ANCOVA, controlling for gender and education years.*

*^c^ANCOVA, controlling for gender, education years and eTIV.*

### Age Effects on Memory Scores

**FIGURE 2:**
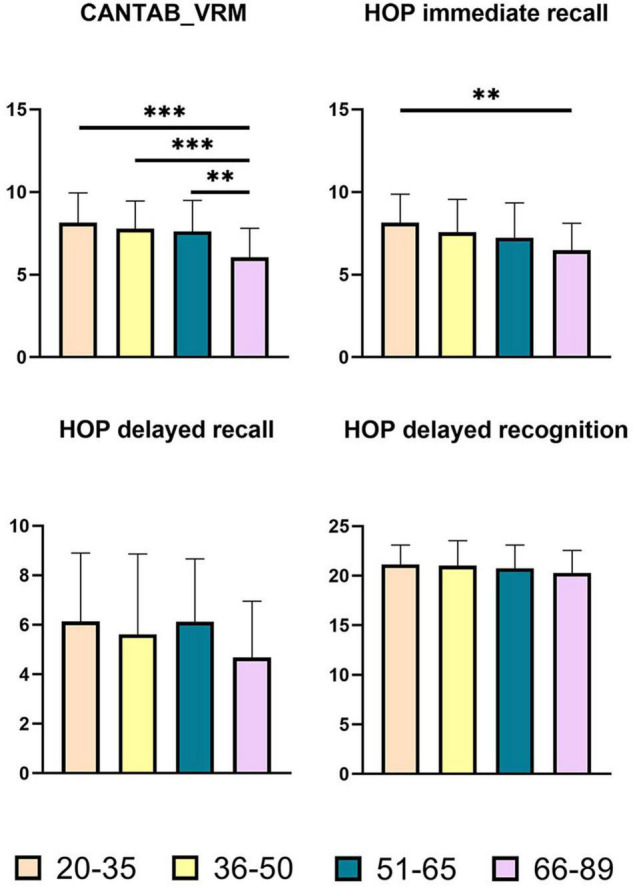
Pairwise comparisons of memory scores between age groups. *X*-axis, age groups; *Y*-axis, memory scores. ^**^P < 0.01, ^***^P < 0.001. CANTAB_VRM, Cambridge Neuropsychological Test Automated Battery verbal recognition memory; HOP immediate recall, Hopkins immediate recall; HOP delayed recall, Hopkins delayed recall; HOP delayed recognition, Hopkins delayed recognition.

The statistical data and ANCOVA analysis results of memory scores are provided given in Table 3. Pairwise comparisons with Bonferroni correction are presented in Figure 2. Results of pairwise comparison are detailed in Supplementary Table 1.

**TABLE 3:** Neuropsychological tests scores.

Characteristics	Young	Middle-early	Middle-late	Old	*F* *^a^*	*P* *^a^*	*F* *^b^*	*P* *^b^*
	(*n* = 29)	(*n* = 23)	(*n* = 34)	(*n* = 63)				
CANTAB_VRM	8.14 ± 1.81	7.78 ± 1.68	7.62 ± 1.88	6.05 ± 1.76	12.630	< 0.001***	12.508	< 0.001***
HOP immediate recall	8.14 ± 1.73	7.57 ± 2.00	7.24 ± 2.10	6.48 ± 1.63	6.116	0.001**	5.953	0.001**
HOP delayed recall	6.14 ± 2.76	5.61 ± 3.26	6.12 ± 2.54	4.68 ± 2.27	3.281	0.023*	3.129	0.028*
HOP delayed recognition	21.14 ± 1.96	21.00 ± 2.54	20.74 ± 2.35	20.29 ± 2.27	1.175	0.322	1.206	0.310

Data expressed as mean ± SD; n.d., not done; *P < 0.05, **P < 0.01, ***P < 0.001.

*^a^ANOVA, no covariates.*

*^b^ANCOVA, controlling for gender and education years. CANTAB_VRM, Cambridge Neuropsychological Test Automatic Battery Verbal Recognition Memory; HOP, Hopkins.*

As shown in Figure 2, there were significant group differences in CANTAB_VRM scores and Hopkins immediate recall scores. Pairwise comparisons further demonstrated apparent declines in CANTAB_VRM scores in the Old group compared with other groups, as well as lower Hopkins immediate recall scores in the Old group compared with the Young group. No significant difference was observed in delayed recall scores or delayed recognition scores. Hopkins delayed recognition scores presented a trend of decrease.

### Age Effects on Amygdala Nuclei Volumes

**FIGURE 3:**
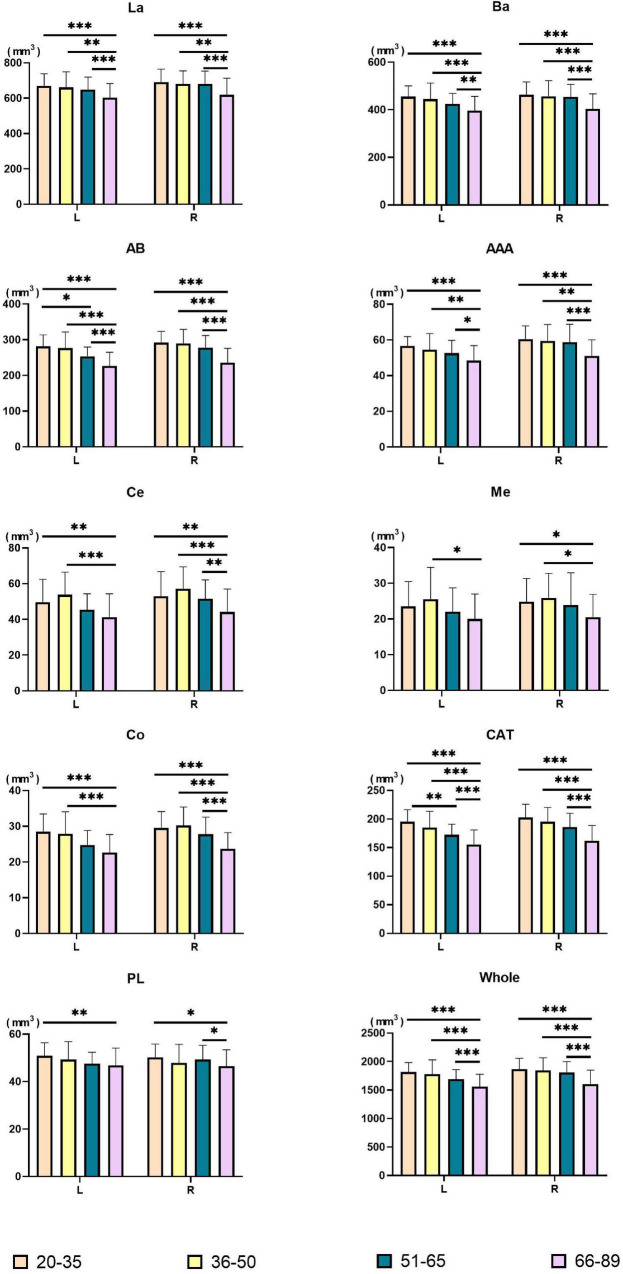
Pairwise comparisons of amygdala nuclei volumes between age groups. *X*-axis, age groups; *Y*-axis, amygdala nuclei volumes. The left and right amygdala were presented in groups. *P < 0.05, ^**^P < 0.01, ^***^P < 0.001. L, left hemisphere; R, right hemisphere; La, lateral nucleus; Ba, basal nucleus; AB, accessory basal nucleus; Ce, central nucleus; Me, medial nucleus; Co, cortical nucleus; AAA, anterior amygdaloid area; CAT, cortico-amygdaloid transition area; PL, paralaminar nucleus; Whole, whole amygdala.

Amygdala nuclei volumes changed with age. Table 4 summarizes the statistical analysis of group differences in amygdala nuclei volumes. Pairwise comparisons with Bonferroni correction results are shown in Figure 3 and detailed in Supplementary Table 3.

**TABLE 4:** Statistical analysis of amygdala and nuclei volumes among the four groups.

Characteristics		Young	Middle-early	Middle-late	Old	*F* *^a^*	*P* *^a^*	*F* *^b^*	*P* *^b^*
		(*n* = 29)	(*n* = 23)	(*n* = 34)	(*n* = 63)				
L	La	670.62 ± 67.65	661.24 ± 88.74	647.89 ± 71.90	602.48 ± 80.43	6.913	< 0.001***	12.951	< 0.001***
	Ba	454.94 ± 8.91	443.28 ± 10.05	433.55 ± 8.31	391.70 ± 6.11	9.423	< 0.001***	14.654	< 0.001***
	AB	281.87 ± 31.95	276.74 ± 45.04	253.86 ± 26.03	226.21 ± 38.87	20.904	< 0.001***	26.610	< 0.001***
	AAA	56.61 ± 5.27	54.60 ± 9.05	52.54 ± 7.32	48.57 ± 8.29	8.548	< 0.001***	10.290	< 0.001***
	Ce	49.80 ± 12.63	53.75 ± 12.76	45.35 ± 8.97	41.30 ± 13.03	7.290	< 0.001***	7.966	< 0.001***
	Me	23.58 ± 6.86	25.57 ± 8.89	22.02 ± 6.71	19.99 ± 7.00	3.965	0.009**	3.557	0.016*
	Co	28.52 ± 4.93	27.91 ± 6.16	24.79 ± 4.04	22.64 ± 5.03	12.075	< 0.001***	12.529	< 0.001***
	CAT	195.60 ± 20.85	185.00 ± 28.73	172.30 ± 18.74	155.45 ± 25.43	21.970	< 0.001***	27.373	< 0.001***
	PL	50.84 ± 5.54	49.27 ± 7.63	47.58 ± 4.82	46.77 ± 7.37	2.844	0.040*	4.670	0.004**
	Whole	1812.88 ± 169.66	1778.84 ± 252.17	1690.57 ± 169.50	1559.36 ± 217.82	13.078	< 0.001***	20.600	< 0.001***
R	La	689.98 ± 74.72	680.54 ± 74.79	681.08 ± 72.39	620.40 ± 93.32	7.228	< 0.001***	11.405	< 0.001***
	Ba	463.74 ± 52.90	456.78 ± 65.65	454.29 ± 52.46	403.76 ± 62.85	10.354	< 0.001***	14.993	< 0.001***
	AB	291.74 ± 31.83	290.12 ± 39.06	278.63 ± 33.32	235.81 ± 40.20	23.161	< 0.001***	29.385	< 0.001***
	AAA	60.53 ± 7.39	59.53 ± 9.19	58.75 ± 10.07	51.11 ± 8.97	10.905	< 0.001***	12.498	< 0.001***
	Ce	52.87 ± 13.92	57.23 ± 12.21	51.50 ± 10.65	44.15 ± 12.88	7.732	< 0.001***	9.693	< 0.001***
	Me	24.84 ± 6.51	25.92 ± 6.86	23.84 ± 9.11	20.54 ± 6.36	4.529	0.005**	4.991	0.003**
	Co	29.57 ± 4.54	30.24 ± 5.15	27.77 ± 4.79	23.69 ± 4.53	17.247	< 0.001***	20.583	< 0.001***
	CAT	202.77 ± 23.16	195.70 ± 24.61	185.73 ± 24.31	161.97 ± 26.94	22.007	< 0.001***	27.790	< 0.001***
	PL	50.12 ± 5.71	47.99 ± 7.72	49.26 ± 6.00	46.50 ± 6.94	2.454	0.066	4.721	0.004**
	Whole	1866.16 ± 190.55	1844.05 ± 222.18	1810.86 ± 187.08	1607.93 ± 241.98	13.894	< 0.001***	20.263	< 0.001***

Data expressed as mean ± SD; n.d., not done; *P<0.05, **P<0.01, ***P < 0.001.

*^a^ANOVA test, no covariates.*

*^b^ANCOVA test, controlling for gender, education years and eTIV. L, left hemisphere; R, right hemisphere; La, lateral nucleus; Ba, basal nucleus; AB, accessory basal nucleus; Ce, central nucleus; Me, medial nucleus; Co, cortical nucleus; AAA, anterior amygdaloid area; CAT, cortico-amygdaloid transition area; PL, paralaminar nucleus; Whole, whole amygdala.*

Volumes of the bilateral La, Ba, AB, AAA, CAT, whole amygdala, left Co, and left PL declined with age. The bilateral Ce, Me, and right Co presented an inverted U shape, with the largest volume in the Middle-early group. Right PL volume decreased in the Middle-early group and the Old group but increased in the Middle-late group. The Volume of the bilateral whole amygdala declined consistently with age, and the volume in the Old group declined significantly (*P* < 0.001) compared with the other three groups. The volumetric changes of most amygdala nuclei were similar to those of the whole amygdala, including the bilateral La, Ba, AB, AAA, CAT, as well as the right Ce and right Co (*P* < 0.05). In addition, a significant decrease in left AB and left CAT volumes was observed in the Middle-late group, earlier than in the whole amygdala. The volumes of the left Ce, right Me, and left Co decreased in the Old group compared with the Young and Middle-early groups. The volume of the left PL declined in the Old group compared with the Young group, and that of the right PL decreased in the Old group compared with the Young and Middle-late groups. Additionally, the left Me volume decreased in the Old group compared with the Middle-early group.

### Associations Between Immediate Memory Function and Amygdala Nuclei Volumes

**FIGURE 4:**
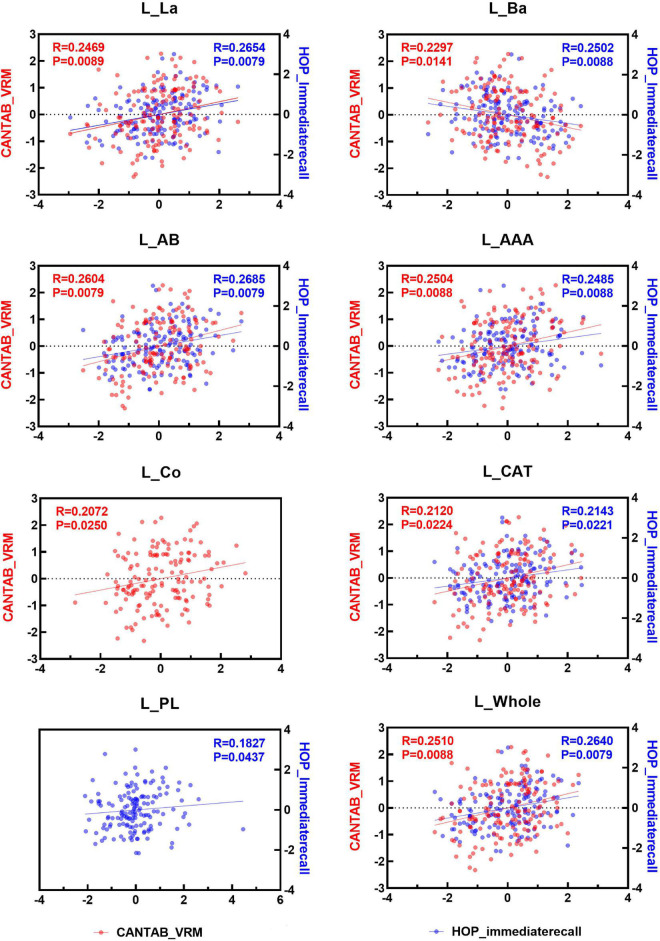
Scatter plots of the Spearman partial correlation between amygdala nuclei volumes and CANTAB_VRM, HOP_immediaterecall scores in the left hemisphere. Only results with significance (*P* < 0.05) were presented. R, correlation coefficient; P, *p*-value of the partial correlation analysis; La, lateral nucleus; Ba, basal nucleus; AB, accessory basal nucleus; Ce, central nucleus; Me, medial nucleus; Co, cortical nucleus; AAA, anterior amygdaloid area; CAT, cortico-amygdaloid transition area; PL, paralaminar nucleus; Whole, whole amygdala; CANTAB_VRM, Cambridge Neuropsychological Test Automatic Battery Verbal Recognition Memory; HOP_immediaterecall, Hopkins immediate recall.

**FIGURE 5:**
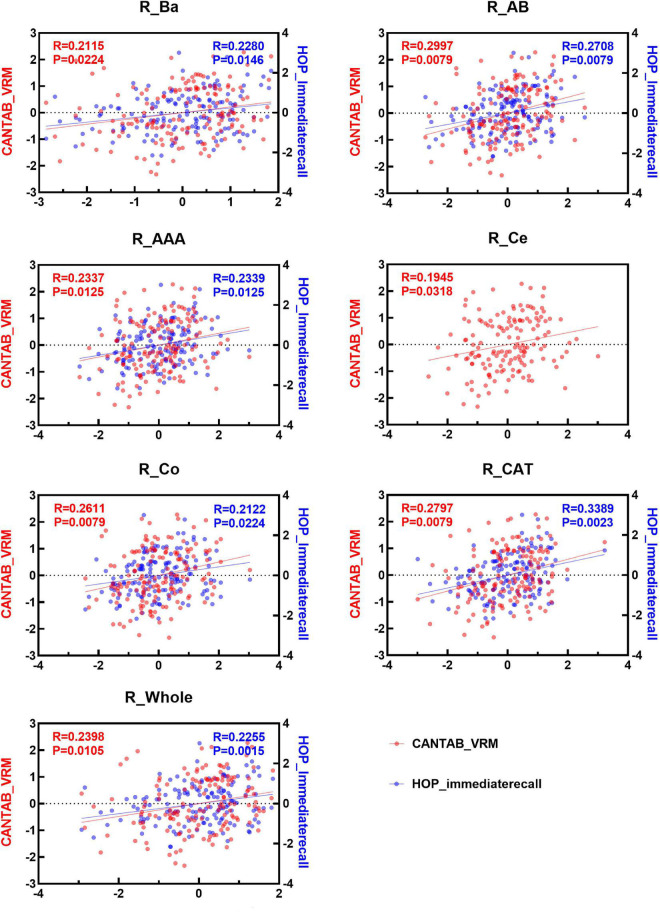
Scatter plots of the Spearman partial correlation between amygdala nuclei volumes and CANTAB_VRM, HOP_immediaterecall scores in the right hemisphere. Only results with significance (*P* < 0.05) were presented. R, correlation coefficient; P, *p*-value of the partial correlation analysis; La, lateral nucleus; Ba, basal nucleus; AB, accessory basal nucleus; Ce, central nucleus; Me, medial nucleus; Co, cortical nucleus; AAA, anterior amygdaloid area; CAT, cortico-amygdaloid transition area; PL, paralaminar nucleus; Whole, whole amygdala; CANTAB_VRM, Cambridge Neuropsychological Test Automatic Battery Verbal Recognition Memory; HOP_immediaterecall, Hopkins immediate recall.

Spearman correlation analysis was applied to examine the association between the two immediate recall memory scores, CANTAB_VRM scores and Hopkins immediate recall scores, and the amygdala nuclei volumes. As shown in Figures 4, 5, both CANTAB_VRM scores and Hopkins immediate recall scores were significantly correlated with the bilateral Ba, AB, AAA, CAT, and whole amygdala as well as the left La and right Co (*P* < 0.05). CANTAB_VRM scores were also correlated with the left Co and right Ce (*P* < 0.05). Hopkins immediate recall scores were also correlated with the left PL (*P* < 0.05). Details of the Spearman correlation were presented in Supplementary Table 4. The mediation analysis revealed no significant mediation effect for the hippocampus on the relationship between amygdala and memory. Details could be found in Supplementary Table 5.

## Discussion

In this cross-sectional study of cognitively normal ApoE ε3/ε3 carriers, we explored the volumetric changes of the whole amygdala and the amygdala nuclei across the adult lifespan, as well as the correlations between neuroimaging and memory performance.

Except the left Me, whose volume decreased in the Old group compared with the Middle-early group, all other nuclei volumes presented significant decline in the Old group compared with the Young group, though they differed in trajectory. The left AB and left CAT volumes were already decreased in the middle-late group. Immediate recall memory scores declined significantly in the Old group compared with the Young group, while no significant alteration was observed in delayed recall or delayed recognition. The decline of immediate recall memory scores was associated with the volumes of the bilateral whole amygdala, Ba, AB, AAA, and CAT and the left La and right Co (*P* < 0.05).

### Age Effects on Amygdala Nuclei Volumes

We observed that in ApoE ε3/ε3 allele carriers, the total amygdala volume declined with age and decreased significantly in the Old group compared with the other groups. Similarly, previous research (Aghamohammadi-Sereshki et al., 2019) found that ApoE ε3/ε3 allele carriers presented significant non-linear age-related volumetric decline—supporting our finding that for ApoE ε3/ε3 allele carriers, the amygdala volume is negatively affected by the aging process.

Other studies of amygdala volume failed to consider the ApoE genotype, and the results were inconsistent with each other. Amygdala volume was observed to decline with age in some neuroimaging studies (Fjell et al., 2009; Narvacan et al., 2017; Wang et al., 2019) but presented no significant age-related change in other studies (Frodl et al., 2008; Cherbuin et al., 2011; Jiang et al., 2014; Wegiel et al., 2017). As the effect of age on amygdala volume is affect by ApoE genotype (Aghamohammadi-Sereshki et al., 2019), this inconsistency might be explained by the difference in ApoE genotype inclusion, highlighting the necessity to consider the ApoE genotype when studying age-related volumetric changes of the amygdala.

Furthermore, we found that except the left Me, whose volume decreased in the Old group compared with the Middle-early group, all other nuclei volumes presented a significant decline in the Old group compared with the Young group, though the trajectories differed among nuclei. Similar to our results, a previous study (Aghamohammadi-Sereshki et al., 2019) found significant non-linear age-related volumetric decline in the La, Ba, AB, and Co but not in the centromedial nucleus in ApoE ε3/ε3 allele carriers. Kurth et al. (2019) described significant negative correlations between age and the volume of the centromedian, laterobasal, and superficial nuclei; they also observed that the decline accelerated with age.

In addition, it is worth noting that for the left AB and left CAT nuclei, the volumetric decrease was observed not only in the Old group but also in the Middle-late group, while volumes of the other nuclei and the whole amygdala only decreased in the Old group. This indicates that the left AB and left CAT might be more sensitive to the aging process than other nuclei and the whole amygdala and suggests that these two nuclei could be used as early neuroimaging biomarkers for age-related changes. Volumes of bilateral Ce, Me, and right Co present the inverted U shape, with the largest volume in the Middle-early group. A similar quadratic trajectory was reported for left superficial nuclei of the amygdala (Kurth et al., 2019).

Compared with previous amygdala nuclei volumetric studies, which segmented the amygdala into three (Bzdok et al., 2013; Kurth et al., 2019) or five (Aghamohammadi-Sereshki et al., 2019) nuclei, the present study achieved greater spatial specificity by studying nine nuclei. Nevertheless, caution should be paid when comparing reports, because the segmentation atlas has varied among studies. Further scrutiny of atlas consistency is required.

### Associations Between Immediate Recall Memory and Amygdala Nuclei

Our analysis revealed a significant decline of immediate recall memory in the Old group compared with the Young group, in accordance with our previous study using the same cohort but without ApoE genotype filtering (Zheng et al., 2018). Age-related immediate recall memory decline was also found in other studies (Ronnlund et al., 2005; Kramer et al., 2007; Josefsson et al., 2012; Nyberg, 2017; Golchert et al., 2019; Rhodes et al., 2019). As ApoE ε4 carrier status is negatively related to immediate free recall memory scores (Golchert et al., 2019), this consistency demonstrates that immediate recall memory function declines in the aging process, even after excluding the confounding factor of ApoE ε4 carrier status. Furthermore, in our cohort, the reduction happened only in the Old group and not in the younger groups, similar to previous reports (Ronnlund et al., 2005; Nyberg, 2017), which found significant episodic memory reduction only after the age of 60, indicating possibly preserved immediate memory function in middle age.

In the present study, we found no significant age-related change in either delayed recall or delayed recognition memory function. However, our previous study using the same cohort but without ApoE genotype filtering (Zheng et al., 2018) reported that delayed recall memory declined in the Old group compared with the Young and Middle-late groups. This inconsistency is derived from the fact that the present research studied only ApoE ε3/ε3 allele carriers, while our previous study (Zheng et al., 2018) also included subjects carrying ApoE ε2 or ε4 alleles. The inconsistency indicates a significant effect of ApoE genotype on age-related alteration of delayed recall memory function, in accordance with a longitudinal study (Golchert et al., 2019) that found that ApoE ε4 carrier status is an important risk factor for delayed free recall memory decline.

Moreover, other studies reported significant decline in delayed free recall with age (Davis et al., 2003) and recognition memory (Rhodes et al., 2019). However, none of the previous studies excluded ApoE ε2 or ε4 allele carriers. Previous studies have demonstrated that ApoE ε4 carrier status is risk factor for age-related memory decline (Caselli et al., 2007; Josefsson et al., 2012; Golchert et al., 2019) in a dose-dependent manner (Caselli et al., 2007) and negatively related to immediate free recall and delayed free recall scores (Golchert et al., 2019). Hence, the previously reported age-related decline of delayed recall and delayed recognition memory function might have resulted from the inclusion of ApoE ε4 allele carriers in the study cohorts. As discussed before, ApoE ε3/ε3 allele carriers are better representative of age-related memory in normal subjects than cohorts including all ApoE genotypes; thus, we conclude that delayed recall and delayed recognition memory functions are preserved in the aging process. The inconsistencies also highlight the necessity of considering ApoE genotype when studying age-related changes of delayed recall and delayed recognition memory function. In addition, subjects in the present cohort were characterized by high education years (16.25 ± 2.24 years), which help preserve memory function in later life (Habib et al., 2007; Golchert et al., 2019).

In our cohort, delayed recall and delayed recognition memory functions represented no significant age-related change in aging, while previous studies have revealed an abnormal decline of these two functions in various dementia disorders. Delayed recall memory function has been reported to decline in conditions such as AD and mild cognitive impairment (Olson et al., 2021), Huntington’s disease (Zakzanis, 1998b), Parkinson’s disease (Higginson et al., 2005), and fronto-temporal dementia (Zakzanis, 1998a). Delayed recognition memory function was observed to decline in Parkinson’s disease (Higginson et al., 2005). Furthermore, it has been reported that individuals with mild cognitive impairment or subjective memory complaints who do not progress to dementia perform better on delayed recall memory function at baseline compared with individuals who progress to dementia (Prado et al., 2019). This suggests the potential use of these two memory functions as diagnostic or prognostic biomarkers for dementia disorders. Further research is necessary to replicate these findings in other samples and advance our understanding of the effects of ApoE genotype on these two memory functions under both healthy and disorder conditions.

## Limitations

There are several limitations in this study that should be considered. All data included in this study were acquired from the DLBS dataset, so we were unable to obtain more clinical information (body mass index, chronic diseases, smoking, alcohol abuse, and lifestyles), which may have influenced our results. At the same time, the DLBS indicated that all participants were healthy adults, but it did not specify the excluding criteria in the subject screening procedure. Therefore, we cannot rule out that our results were affected at least to some extent by these factors.

## Conclusion

To our knowledge, this is the first study to relate immediate recall memory to the amygdala and its nuclei on ApoE ε3/ε3 allele carriers. Our findings suggest that immediate recall is impaired by aging. Furthermore, the volumetric decrease of the bilateral Ba, AB, AAA, and CAT nuclei, whole amygdala, and left La and right Co associated with the reduction of immediate recall memory function. The present study highlights the left AB and left CAT might be considered as potential imaging biomarkers of memory decline in aging.

## Data Availability Statement

The raw data supporting the conclusions of this article will be made available by the authors, without undue reservation.

## Ethics Statement

The studies involving human participants were reviewed and approved by the Institutional Review Board of the University of Texas Southwestern Medical Center and the University of Texas at Dallas. The patients/participants provided their written informed consent to participate in this study.

## Author Contributions

WqL and DC designed the study. WqL performed the statistical analysis and prepared the original draft. DC collected the data, performed the neuroimage processing, and reviewed and edited the draft. TY, ZL, JJ, WbL, HW, and XW reviewed the manuscript. All the authors read and approved the submitted manuscript.

## Conflict of Interest

WbL was employed by Sinovation (Beijing) Medical Technology Co., Ltd. The research was not sponsored by the company, and the data was from a public database. The remaining authors declare that the research was conducted in the absence of any commercial or financial relationships that could be construed as a potential conflict of interest.

## Publisher’s Note

All claims expressed in this article are solely those of the authors and do not necessarily represent those of their affiliated organizations, or those of the publisher, the editors and the reviewers. Any product that may be evaluated in this article, or claim that may be made by its manufacturer, is not guaranteed or endorsed by the publisher.

## Footnotes

1
http://fcon_1000.projects.nitrc.org/indi/retro/dlbs.html

2
http://surfer.nmr.mgh.harvard.edu/
