# Corrigendum: Correlation between amygdala nuclei volumes and memory in cognitively normal adults carrying the ApoE ε3/ε3 allele

**DOI:** 10.3389/fnagi.2022.1087156

**Published:** 2022-11-21

**Authors:** Wenqing Liao, Dong Cui, Jingna Jin, Wenbo Liu, Xin Wang, He Wang, Ying Li, Zhipeng Liu, Tao Yin

**Affiliations:** ^1^Institute of Biomedical Engineering, Chinese Academy of Medical Sciences and Peking Union Medical College, Tianjin, China; ^2^Sinovation (Beijing) Medical Technology Co., Ltd., Beijing, China; ^3^Neuroscience Center, Chinese Academy of Medical Sciences, Beijing, China

**Keywords:** amygdala nuclei, aging, ApoE, immediate recall, delayed recall, delayed recognition

In the published article, there was an error in Table 2 as published. The compartment unit was displayed as “mm^3^,” when it should be “cm^3^.” The corrected Table 2 appears below.

**Table 2:** Cerebral compartment volumes.

**Compartment (cm^3^)**	**Young**	**Middle-early**	**Middle-late**	**Old**	***F* ^a^**	***P* ^a^**	***F*^b^/^c^**	***P*^b^/^c^**
	**(*n* = 29)**	**(*n* = 23)**	**(*n* = 34)**	**(*n* = 63)**				
eTIV	1408.21 ± 17.83	1394.82 ± 20.07	1381.02 ± 16.46	1429.89 ± 12.09	1.608	0.190	2.129^b^	0.099^b^
Gray matter	673.59 ± 9.23	633.32 ± 5.81	607.31 ± 10.36	593.79 ± 8.52	40.88	< 0.001^***^	143.00	< 0.001^***^
White matter	533.74 ± 10.92	539.94 ± 12.26	508.63 ± 10.08	488.61 ± 7.41	6.35	< 0.001^***^	24.49	< 0.001^***^
Cerebro-spinal fluid	224.22 ± 13.58	269.67 ± 15.25	308.46 ± 12.54	429.08 ± 9.21	64.49	< 0.001^***^	103.65	< 0.001^***^

Data expressed as mean ± SD; eTIV, estimated total intracranial volume; n.d., not done;

*** P < 0.001.

a ANOVA, no covariates.

b ANCOVA, controlling for gender and education years.

c ANCOVA, controlling for gender, education years and eTIV.

The authors apologize for this error and state that this does not change the scientific conclusions of the article in any way. The original article has been updated.

## Publisher's note

All claims expressed in this article are solely those of the authors and do not necessarily represent those of their affiliated organizations, or those of the publisher, the editors and the reviewers. Any product that may be evaluated in this article, or claim that may be made by its manufacturer, is not guaranteed or endorsed by the publisher.

