# Supplementary material for: Correlation Between Amygdala Nuclei Volumes and Memory in Cognitively Normal Adults Carrying the ApoE ε3/ε3 Allele
Source: Front Aging Neurosci. 2021 Dec 13;13:747288. doi: 10.3389/fnagi.2021.747288 (PMC8713572; doi:10.3389/fnagi.2021.747288)
Supplement: Supplementary file 1 [file Data_Sheet_1.docx]

Supplementary Material

**Supplementary Table 1** Pairwise comparison of cognitive assessment

| **Cognitive score** | **ANCOVA** | **Pairwise comparison** | | | | | |
| --- | --- | --- | --- | --- | --- | --- | --- |
|  | ***P* value** | ***P1*** | ***P2*** | ***P3*** | ***P4*** | ***P5*** | ***P6*** |
| MMSE | <0.001^***^ | 1.000 | 1.000 | <0.001^***^ | 1.000 | 0.007^**^ | 0.001^**^ |
| CANTAB_VRM | <0.001^***^ | 1.000 | 1.000 | <0.001^***^ | 1.000 | <0.001^***^ | 0.001^**^ |
| HOP immediate recall | 0.001^**^ | 1.000 | 0.276 | 0.001^**^ | 1.000 | 0.065 | 0.454 |
| HOP delayed recall | 0.028^*^ | 1.000 | 1.000 | 0.094 | 1.000 | 0.585 | 0.091 |
| HOP delayed recognition | 0.310 | 1.000 | 1.000 | 0.628 | 1.000 | 0.964 | 1.000 |

Note: *P1*: young vs middle-early; *P2*: young vs middle-late; *P3*: young vs old; *P4*: middle-early vs middle-late; *P5*: middle-early vs old; *P6*: middle-late vs old; **P* < 0.05, ***P* < 0.01, ****P* < 0.001.

Abbreviations: MMSE: Mini-Mental State Examination; CANTAB_VRM: Cambridge Neuropsychological Test Automated Battery verbal recognition memory; HOP: Hopkins.

**Supplementary Table 2** Pairwise comparison of cerebral compartment volumes

| **Cognitive score** | **ANCOVA** | **Pairwise comparison** | | | | | |
| --- | --- | --- | --- | --- | --- | --- | --- |
|  | ***P* value^a/b^** | ***P1^b^*** | ***P2^b^*** | ***P3^b^*** | ***P4^b^*** | ***P5^b^*** | ***P6^b^*** |
| eTIV | 0.099^a^ | n.d. | n.d. | n.d. | n.d. | n.d. | n.d. |
| cerebrospinal fluid | <0.001^***^ | 0.055 | <0.001^***^ | <0.001^***^ | 0.001 | <0.001^***^ | <0.001^***^ |
| grey matter | <0.001^***^ | <0.001^***^ | <0.001^***^ | <0.001^***^ | 0.007 | <0.001^***^ | <0.001^***^ |
| White matter | <0.001^***^ | 1.000 | 1.000 | <0.001^***^ | 1.000 | <0.001^***^ | <0.001^***^ |

Note: n.d., not done; ^a^ANCOVA, controlling for gender and education years; ^b^ANCOVA, controlling for gender, education years and eTIV. *P1*: young vs middle-early; *P2*: young vs middle-late; *P3*: young vs old; *P4*: middle-early vs middle-late; *P5*: middle-early vs old; *P6*: middle-late vs old; **P* < 0.05, ***P* < 0.01, ****P* < 0.001.

Abbreviations: eTIV, evaluated total intracranial volume.

**Supplementary Table 3** Pairwise comparison of amygdala nuclei volume

| **Amygdala nuclei** | | **ANCOVA** | | **Pairwise comparison** | | | | | | |
| --- | --- | --- | --- | --- | --- | --- | --- | --- | --- | --- |
|  |  | ***P* value** | | ***P1*** | ***P2*** | | ***P3*** | ***P4*** | ***P5*** | ***P6*** |
| L | La | | <0.001^***^ | 1.000 | | 1.000 | <0.001^***^ | 1.000 | 0.001^**^ | <0.001^***^ |
|  | Ba | | <0.001^***^ | 1.000 | | 0.488 | <0.001^***^ | 1.000 | <0.001^***^ | 0.001^**^ |
|  | AB | | <0.001^***^ | 1.000 | | 0.043^*^ | <0.001^***^ | 0.417 | <0.001^***^ | <0.001^***^ |
|  | AAA | | <0.001^***^ | 1.000 | | 0.481 | <0.001^***^ | 1.000 | 0.004^**^ | 0.010^*^ |
|  | Ce | | <0.001^***^ | 1.000 | | 1.000 | 0.005^**^ | 0.231 | <0.001^***^ | 0.128 |
|  | Me | | 0.016^*^ | 1.000 | | 1.000 | 0.205 | 1.000 | 0.021^*^ | 0.621 |
|  | Co | | <0.001^***^ | 1.000 | | 0.055 | <0.001^***^ | 0.414 | <0.001^***^ | 0.063 |
|  | CAT | | <0.001^***^ | 0.464 | | 0.003^**^ | <0.001^***^ | 0.802 | <0.001^***^ | <0.001^***^ |
|  | PL | | 0.004^**^ | 1.000 | | 0.888 | 0.003^**^ | 1.000 | 0.206 | 0.306 |
|  | Whole | | <0.001^***^ | 1.000 | | 0.353 | <0.001^***^ | 1.000 | <0.001^***^ | <0.001^***^ |
| R | La | | <0.001^***^ | 1.000 | | 1.000 | <0.001^***^ | 1.000 | 0.005^**^ | <0.001^***^ |
|  | Ba | | <0.001^***^ | 1.000 | | 1.000 | <0.001^***^ | 1.000 | <0.001^***^ | <0.001^***^ |
|  | AB | | <0.001^***^ | 1.000 | | 1.000 | <0.001^***^ | 1.000 | <0.001^***^ | <0.001^***^ |
|  | AAA | | <0.001^***^ | 1.000 | | 1.000 | <0.001^***^ | 1.000 | 0.001^**^ | <0.001^***^ |
|  | Ce | | <0.001^***^ | 1.000 | | 1.000 | 0.003^**^ | 1.000 | <0.001^***^ | 0.003^**^ |
|  | Me | | 0.003^**^ | 1.000 | | 1.000 | 0.031^*^ | 1.000 | 0.016^*^ | 0.055 |
|  | Co | | <0.001^***^ | 1.000 | | 1.000 | <0.001^***^ | 0.905 | <0.001^***^ | <0.001^***^ |
|  | CAT | | <0.001^***^ | 1.000 | | 0.144 | <0.001^***^ | 1.000 | <0.001^***^ | <0.001^***^ |
|  | PL | | 0.004^**^ | 1.000 | | 1.000 | 0.023^*^ | 0.977 | 1.000 | 0.010^*^ |
|  | Whole | | <0.001^***^ | 1.000 | | 1.000 | <0.001^***^ | 1.000 | <0.001^***^ | <0.001^***^ |

Note: *P1*: young vs middle-early; *P2*: young vs middle-late; *P3*: young vs old; *P4*: middle-early vs middle-late; *P5*: middle-early vs old; *P6*: middle-late vs old; **P* < 0.05, ** *P* < 0.01, ****P* < 0.001.

Abbreviations: L, left hemisphere; R, right hemisphere; La: lateral nucleus; Ba: basal nucleus; AB: accessory basal nucleus; Ce: central nucleus; Me: medial nucleus; Co: cortical nucleus; AAA: anterior amygdaloid area; CAT: cortico-amygdaloid transition area; PL: paralaminar nucleus; Whole: whole amygdala.

**Supplementary Table 4** Correlation analysis between amygdala nuclei volumes and memory scores

| **Characteristics** | | **CANTAB_VRM** | | **HOP immediate recall** | |
| --- | --- | --- | --- | --- | --- |
|  |  | ***R*** | ***P*** | ***R*** | ***P*** |
| L | La | 0.2469 | 0.0089** | 0.2654 | 0.0079** |
|  | Ba | 0.2297 | 0.0141* | 0.2502 | 0.0088** |
|  | AB | 0.2604 | 0.0079** | 0.2685 | 0.0079** |
|  | AAA | 0.2504 | 0.0088** | 0.2485 | 0.0088** |
|  | Ce | 0.0951 | 0.2921 | 0.1601 | 0.0765 |
|  | Me | 0.0851 | 0.3458 | 0.0501 | 0.5625 |
|  | Co | 0.2072 | 0.0250* | 0.1488 | 0.0943 |
|  | CAT | 0.2120 | 0.0224* | 0.2143 | 0.0221* |
|  | PL | 0.1725 | 0.0575 | 0.1827 | 0.0437* |
|  | Whole | 0.2510 | 0.0088** | 0.2640 | 0.0079** |
| R | La | 0.1707 | 0.0591 | 0.1478 | 0.0953 |
|  | Ba | 0.2115 | 0.0224* | 0.2280 | 0.0146* |
|  | AB | 0.2997 | 0.0079** | 0.2708 | 0.0079** |
|  | AAA | 0.2337 | 0.0125* | 0.2339 | 0.0125* |
|  | Ce | 0.1945 | 0.0318* | 0.0947 | 0.2921 |
|  | Me | 0.0708 | 0.4276 | 0.1353 | 0.1295 |
|  | Co | 0.2611 | 0.0079** | 0.2122 | 0.0224* |
|  | CAT | 0.2797 | 0.0079** | 0.3389 | 0.0023** |
|  | PL | 0.0470 | 0.5808 | 0.1619 | 0.0741 |
|  | Whole | 0.2398 | 0.0105* | 0.2255 | 0.0015** |

*R*, correlation coefficient. **P* < 0.05, ***P* < 0.01. All data was Z-score normalized.

Abbreviations: L, left hemisphere; R, right hemisphere; La, lateral nucleus; Ba, basal nucleus; AB, accessory basal nucleus; Ce, central nucleus; Me, medial nucleus; Co, cortical nucleus; AAA, anterior amygdaloid area; CAT, cortico-amygdaloid transition area; PL, paralaminar nucleus; Whole, whole amygdala; CANTAB_VRM, Cambridge Neuropsychological Test Automatic Battery Verbal Recognition Memory; HOP immediate recall, Hopkins immediate recall.

**Supplementary Table 5.** Mediation effect analysis results.

| Dependent variable | Independent variable | Mediating variable | Mediation effect | |
| --- | --- | --- | --- | --- |
|  |  |  | coefficient | P value |
| CANTAB_VRM | L Amygdala | L hippocampus | .0004 | .4373 |
|  | R Amygdala | R hippocampus | .0005 | .3298 |
| HOP_immediaterecall | L Amygdala | L hippocampus | .0003 | .6001 |
|  | R Amygdala | R hippocampus | .0003 | .4978 |
| CANTAB_VRM | L hippocampus | L Amygdala | .0018 | .1405 |
|  | R hippocampus | R Amygdala | .0011 | .3641 |
| HOP_immediaterecall | L hippocampus | L Amygdala | .0018 | .1242 |
|  | R hippocampus | R Amygdala | .0015 | .1914 |

Abbreviations: L, left hemisphere; R, right hemisphere; CANTAB_VRM, Cambridge Neuropsychological Test Automatic Battery Verbal Recognition Memory; HOP_immediaterecall, Hopkins immediate recall.


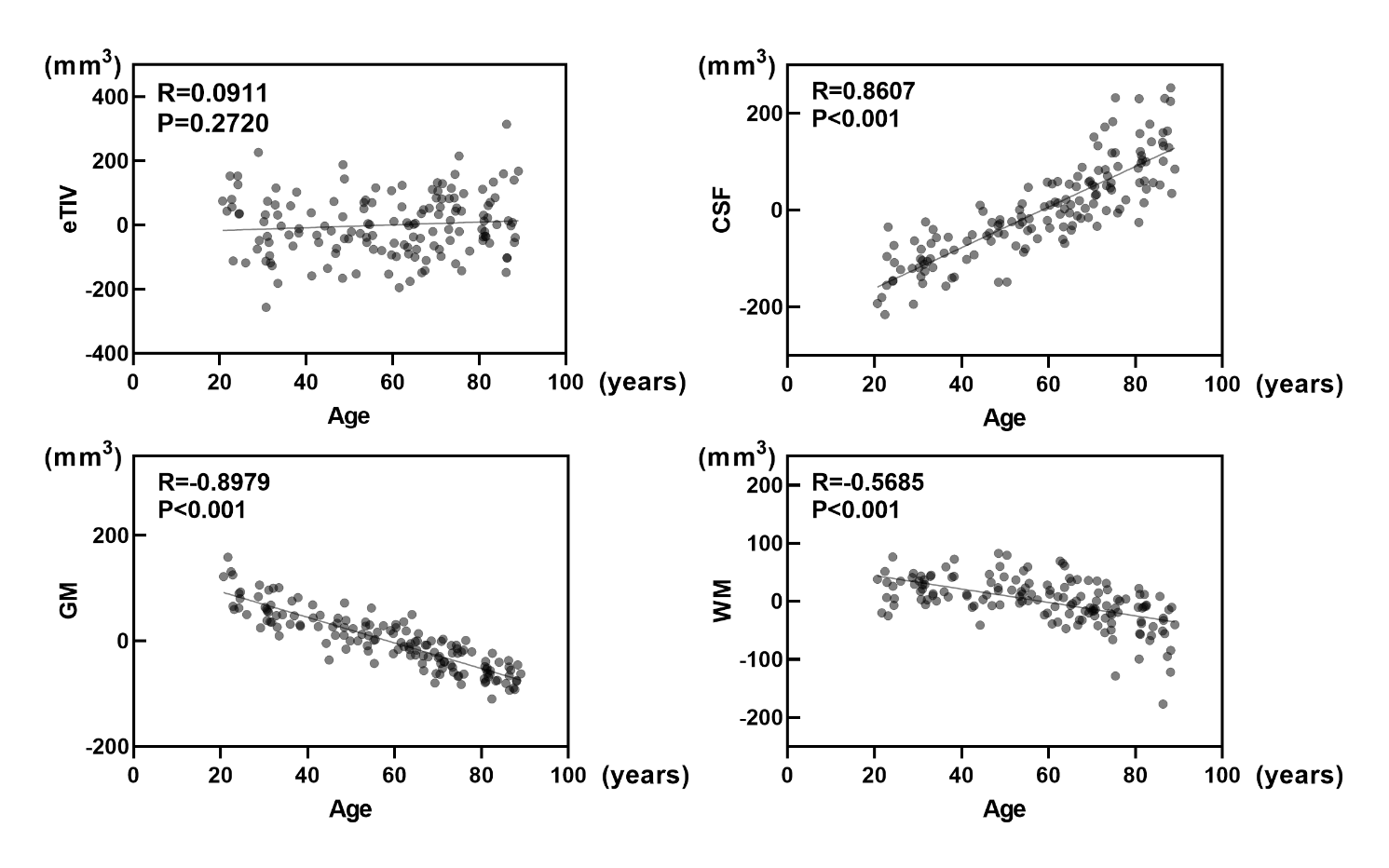
 **Supplementary Figure 1**. Scatter plots of the Pearson partial correlation between cerebral compartments and age.

Abbreviations: eTIV, evaluated total intracranial volume; CSF, cerebrospinal fluid; GM, grey matter; WM, white matter.
